# Antioxidant and Anti-Inflammatory Benefits of *Gymnema inodorum* Leaf Extract in Human Umbilical Vein Endothelial Cells Under Peroxynitrite Stress

**DOI:** 10.3390/antiox14040427

**Published:** 2025-04-01

**Authors:** Onanong Nuchuchua, Suthasinee Seephan, Wanwisa Srinuanchai, Piya Temviriyanukul, Varisa Pongrakhananon

**Affiliations:** 1 National Nanotechnology Center (NANOTEC), National Science and Technology Development Agency (NSTDA), Pathum Thani 12120, Thailand; wanwisa.sri@nanotec.or.th; 2 Department of Pharmacology and Physiology, Faculty of Pharmaceutical Sciences, Chulalongkorn University, Bangkok 10330, Thailand or sutha@inm.u-toyama.ac.jp (S.S.); varisa.p@pharm.chula.ac.th (V.P.); 3 Institute of Natural Medicine, University of Toyama, Toyama 930-0194, Japan; 4 Institute of Nutrition, Mahidol University, Nakhon Pathom 73170, Thailand; piya.tem@mahidol.ac.th; 5 Center of Excellence in Preclinical Toxicity and Efficacy Assessment of Medicines and Chemicals, Chulalongkorn University, Bangkok 10330, Thailand

**Keywords:** *Gymnema inodorum* leaf extract, peroxynitrite, oxidative stress, human umbilical vein endothelial cells, gene expression, enzymatic antioxidants, anti-inflammatory cytokine responses

## Abstract

Endothelial dysfunction driven by oxidative and nitrosative stress is a critical factor in the pathogenesis of diabetes-related vascular complications. This study investigated the antioxidant and anti-inflammatory effects of *Gymnema inodorum* leaf (GiL) extract and its flavonoid constituents, kaempferol and quercetin, on human umbilical vein endothelial cells (HUVECs) exposed to peroxynitrite-induced stress. Peroxynitrite exposure significantly reduced the mRNA levels of antioxidant enzymes (e.g., catalase, glutathione peroxidase 1, superoxide dismutase 1, and superoxide dismutase 2) while increasing the expression of pro-inflammatory cytokines (e.g., tumor necrosis factor-alpha, interleukin-1 beta, interleukin-6, interleukin-10, and interleukin-12), ultimately leading to oxidative stress and cellular damage. Treatment with GiL extract reversed these effects by enhancing the defenses of antioxidants through the upregulation of enzymatic mRNA expression and suppressing inflammation via the downregulation of cytokine gene expression. The flavonoid constituents of the extract were identified as the active compounds responsible for these protective effects, with kaempferol and quercetin exhibiting significant free radical scavenging activity and the modulation of inflammatory signaling pathways. High doses of GiL extract showed greater efficacy in restoring cellular homeostasis and preventing oxidative damage. These findings underscore the potential of *Gymnema inodorum* as a source of bioactive compounds for preventing and managing endothelial dysfunction and other oxidative stress-related complications in diabetes.

## 1. Introduction

Oxidative and nitrosative agents are produced at high levels in individuals with type 2 diabetes mellitus (T2DM). The mitochondrial respiratory chain, glycolysis, and glucose autoxidation that characterize this disease generate reactive oxygen species (ROS) as a result of prolonged hyperglycemia [1,2]. ROS can induce abnormality in the glycolysis pathway, resulting in the generation of advanced glycation end products (AGEs) and apoptosis [3,4]. Furthermore, excess ROS can spontaneously react with nitric oxide produced by *endothelial* vascular cells to generate highly reactive nitrogen species (RNS) such as peroxynitrite [5]. This reaction lowers the production of nitric oxide by endothelial vascular cells. As a result, the endothelial cells lose their biological ability to produce vasoactive substances, hormones, and cytoprotective biological factors [6,7]. Micro- and macrovascular systemic problems later develop, inducing and increasing the severity of diabetic complications, e.g., neuropathy, nephropathy, retinopathy, and chronic wounds [8,9].

Several studies have shown that patients with diabetes have low levels of plasma antioxidants [10]. It is recommended for patients with diabetes to consume foods that are high in antioxidants to reduce oxidative stress and reverse vascular diabetic complications. The dietary antioxidants recommended for diabetes include carotenoids, flavonoids, polyphenols, glutathione, polyamines, and vitamins A, C, and E [11,12,13]. These compounds are involved in antioxidant scavenging systems and can assist in the neutralization of free radicals produced via diabetic metabolism [14].

Exogenous antioxidants can also be obtained from herbs, fruits, and vegetables. Our research has focused on the antidiabetic-promoting benefits of *Gymnema inodorum*, a leafy green vegetable found mainly in northern Thailand [15,16,17]. *G. inodorum* leaf extract (GiL extract) has been prepared via solvent maceration and used as an anti-diabetic medication by the Thai Yuan ethnic group since ancient times [17]. GiL extract contains vitamins, carotenes, xanthophylls, tannins and phenolics, triterpene saponins, and pregnane glycosides [18]. The phytochemicals found in this extract can control diabetic mellitus by regulating digestive enzymes [19,20], the intestinal glucose uptake [16,21], and insulin-mimetic activity [15,22].

GiL extract is also an antioxidant and anti-inflammatory ingredient [15,23,24,25,26]. The antioxidant activities of *G. inodorum* are associated with free radical scavenging mechanisms via hydrogen atom transfer (HAT) and single-electron transfer (SET). The analysis of data on GiL using methods such as Pearson’s correlation coefficients, principal component analysis (PCA), and hierarchical cluster analysis (HCA) showed that the scavenging activities of *G. inodorum* correspond to its flavonoid contents, specifically to kaempferol and quercetin [23]. The high content of flavonoids in GiL extracts had a significant antioxidant effect on human umbilical vein endothelial cells (HUVECs) upon exposure to peroxynitrite [23]. Moreover, oxidative cells pre-treated with *G. inodorum* leaf extracts showed more viability than untreated cells [23]. Anti-inflammatory effects of GiL extract were also observed in macrophages following lipopolysaccharide (LPS)-induced inflammation. GiL extract reduced the production of nitic oxide (an inflammatory signal) by LPS-induced macrophages [15]. However, the anti-inflammatory effects of GiL extract have not been studied in HUVECs.

This study aimed to investigate the antioxidant and anti-inflammatory effects of GiL extract on HUVECs exposed to nitrosative stress by peroxynitrite to assess the protective vascular effects of GiL phytonutrients in individuals with diabetes. The cellular observation conducted in this study involved the analysis of the mRNA expression, including that of antioxidant enzymes (e.g., catalase (*CAT)*, glutathione peroxidase 1 (*GPX1*), superoxide dismutase 1 (*SOD1)*, and superoxide dismutase 2 (*SOD2)*) and inflammatory and anti-inflammatory response cytokines (e.g., tumor necrosis factor-alpha (*TNF-α*), interleukin-1 beta (*IL-1β*), interleukin-6 (*IL-6)*, interleukin-10 (*IL-10)*, and interleukin-12 (*IL-12)*) of the treated HUVECs.

## 2. Materials and Methods

### 2.1. Preparation of Gymnema inodorum Leaf (GiL) Extract

*G. inodorum* leaf sample was purchased from the Chiang-Da farming group, Fang District, Chiang Mai Province, Thailand [17,26]. The leaf sample was washed with water and dried in a hot-air oven at 60 °C. GiL crude extract was prepared using the extraction protocol described by Srinuanchai et al., 2020 [20]. Briefly, the dried leaf matter (250 g) was ground and soaked in 2.5 L of ethanol (75% *v*/*v*). The solution was filtered through a paper filter and the GiL extract solution was obtained after ethanol removal. The aqueous solution was lyophilized to obtain powdered GiL extract. The pigments and fat in the GiL extract were discarded via fractionation using a mixture of hexane, ethyl acetate, methanol, and water at a volume ratio of 1:1:1:1 [27]. The lower part of the solvent fraction was collected and made into a powder for further analysis. The GiL extract used in this study comprised 1.4% *w*/*w* kaempferol and 2.1% *w*/*w* quercetin.

### 2.2. Quantification of Kaempferol and Quercetin Using High-Performance Liquid Chromatography (HPLC)

Kaempferol and quercetin were quantified via acid hydrolysis following the protocol of Sirichai et al. [28]. Chromatographic analysis of the sample solution was conducted using a Waters chromatograph (Milford, MA, USA) equipped with an Alliance 2695 separation module and a 2998 photodiode array detector. The results were analyzed using Empower Pro 2 Software (Waters, MA, USA). Briefly, 20 µL of a standard mixture solution containing kaempferol (0–100 µg/mL), quercetin (0–100 µg/mL), and the GiL extract (1000 µg/mL) were injected into a reverse phase column (Luna^®^ C_18_ 100 Å 150 mm × 4.6 mm) connected to a SecurityGuard^TM^ C_18_ with dimensions of 4 mm × 2.0 mm from Phenomenex, Inc., Bangkok, Thailand. Chemical separation was performed using two mobile phases: solution A (0.1% *v*/*v* formic acid in acetonitrile) and solution B (0.1% *v*/*v* formic acid in deionized water). Formic acid and acetonitrile (LiChrosolv^®^) were obtained from Merck, Darmstadt, Germany. A gradient elution was performed from 5% A to 90% A over 40 min; 90% A was held for 5 min before the solution was changed to 5% A over 5 min and then held at that percentage for 5 min. The total running time was 55 min. The flow rate of the system was 0.6 mL/min. Kaempferol and quercetin absorbances were measured at 264 nm.

### 2.3. Human Umbilical Vein Endothelial Cell Culture

Human umbilical vein endothelial cells (HUVECs) were obtained from ATCC-CRL-1730^™^ (ATCC, Manassas, VA, USA). The cells were cultured following a protocol developed by ATCC using a complete F-12K medium containing supplementary components (0.1 mg/mL of heparin, 1% *v*/*v* 10,000 unit/mL of penicillin–streptomycin, 10% *v*/*v* FBS, and 30 µg/mL of endothelial cell growth supplement). The cells were cultured at 37 °C with 5% CO_2_. After the cell confluency reached 90%, HUVECs were seeded into a 96-well plate at 2 × 10^4^ cells/well for cytotoxicity assay and into a 6-well plate at 3 × 10^5^ cells/well for gene expression analysis. M199 medium (no phenol red) (Thermo Fisher Scientific Inc., Waltham, MA, USA) was used instead of the F-12K medium for cell-based analysis.

#### 2.3.1. Cytotoxic Studies of Peroxynitrite and Tested Compounds

Peroxynitrite (0–500 µM) (Merck, Darmstadt, Germany) and tested compounds (GiL extract, kaempferol, and quercetin (TCI, Tokyo, Japan) at 0–250 µg/mL) were evaluated for cytotoxicity against HUVECs over 48 h. Cell viability was assessed using the MTS assay (CellTiter 96^®^ AQueous One Solution Cell Proliferation Assay, Promega Corporation, Madison, WI, USA), where viable cells reduced tetrazolium salts to produce a purple formazan dye; absorbance was measured at 490 nm. The peroxynitrite concentration causing 20% cell death was selected as the active dose for evaluating the protective effects of the GiL extract.

#### 2.3.2. Screening for Protective Doses of Tested Compounds

HUVECs were freshly seeded in a 96-well plate at 2 × 10^4^ cells/well and left overnight at 37 °C and 5% CO_2_ for cell stabilization. Non-toxic doses of the tested compounds were pre-incubated for 24 h, followed by 1 h exposure to peroxynitrite (14.5 µM). Then, the medium was removed and replaced with the MTS solution, and cell recovery was determined based on absorbance at 490 nm.

### 2.4. Quantitative Reverse Transcription–Polymerase Chain Reaction (qRT-PCR) Analysis

HUVECs (3 × 10^5^ cells/well) were seeded in 6-well plates and incubated overnight at 37 °C and 5% CO_2._ The cells were treated with mixtures with different concentrations of kaempferol and quercetin (KQ) or GiL extract for 24 h, which was followed by 1 h exposure to peroxynitrite. The cells were harvested, centrifuged (2000× *g*, 4 °C, 5 min), and lysed with 1 mL GENEzol™ reagent (Geneaid Biotech, New Taipei City, Taiwan). RNA was extracted using chloroform, purified with isopropanol, washed with 75% ethanol, and centrifuged at 12,000× *g*. The RNA pellet was dehydrated at room temperature for 30 min. Total RNA was quantified using a Nanodrop spectrophotometer (Thermo Fisher Scientific, MA, USA), and 1 µg RNA was reverse transcribed to cDNA using ProtoScript II Reverse transcriptase (Invitrogen, MA, USA). PCR amplification using a SensiFAST™ SYBR Green Supermix (SensFAST^TM^ SYBR Green Supermix (Bioline, TN, USA) and a StepOne™ RT PCR system (Thermo Fisher Scientific, MA, USA) included the following steps: denaturation at 95 °C for 30 s; 40 cycles of melting at 95 °C for 5 s, annealing at 57–62 °C for 30 s, elongation at 72 °C for 30 s; and final extension at 72 °C for 10 min. Relative gene expression was calculated using the 2^−ΔΔct^ method [29]. The primers used are listed in Table 1. Each experiment was repeated three times for accuracy and reliability.

### 2.5. Statistical Analysis

One-way ANOVA with Dunnett’s post-hoc test was conducted using GraphPad Prism 8.0 (GraphPad Software, Boston, MA, USA). Statistically significant differences were determined based on *p*-values: * and ** represent *p ≤* 0.05 and *p ≤* 0.01, respectively, compared with the normal HUVECs, while # and ## represent *p ≤* 0.05 and *p ≤* 0.01, respectively, compared with cells subjected to nitrosative/oxidative stress.

## 3. Results

### 3.1. Cytotoxicity of Peroxynitrite and Tested Compounds

Peroxynitrite and the tested compounds (GiL extract, kaempferol, and quercetin) were evaluated to determine their cytotoxic and non-cytotoxic doses for HUVECs. An increase in the peroxynitrite concentration gradually decreased the viability of the HUVECs. Peroxynitrite concentrations lower than 3.5 µM did not cause cell death, while concentrations up to 14.5 µM killed 20% of the cells (Supplementary Material, Figure S1). The GiL extract solutions at concentrations of 0–125 µg/mL were not toxic to the HUVECs (Supplementary Material, Figure S2), while kaempferol and quercetin were non-toxic at concentrations up to 31.2 µg/mL and 62.5 µg/mL, respectively (See Supplementary Material Figures S3 and S4).

In this study, 3.5 µM and 14.5 µM concentrations of peroxynitrite were used as low and high doses to study the nitrosative stress exerted on HUVECs by these concentrations. They were marked as L-ONOO^−^ and H-ONOO^−^, respectively. Furthermore, the effects of the GiL extract and the kaempferol and quercetin mixture (KQ) on the HUVECs and the nitrosative cells were monitored. Non-toxic doses of the GiL extract, 62.5 µg/mL (1x_GiL) and 125 µg/mL (2x_GiL), were used. The equivalent KQ mixed solution for 1x_GiL was 1x_KQ (containing 0.9 µg/mL of kaempferol and 1.3 µg/mL of quercetin), while that for 2x_GiL was 2x_KQ, comprising 1.8 µg/mL of kaempferol and 2.6 µg/mL of quercetin.

### 3.2. Effects of Peroxynitrite on HUVECs

**Figure 1:**
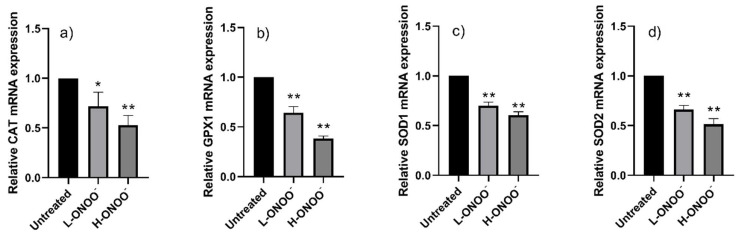
Relative mRNA expressions of (**a**) *CAT*, (**b**) *GPX1*, (**c**) *SOD1*, and (**d**) *SOD2* in HUVECs after incubation with peroxynitrite at low dose (L-ONOO^−^) and high dose (H-ONOO^−^). * *p* ≤ 0.05 and ** *p* ≤ 0.01 vs. untreated HUVECs.

**Figure 2:**
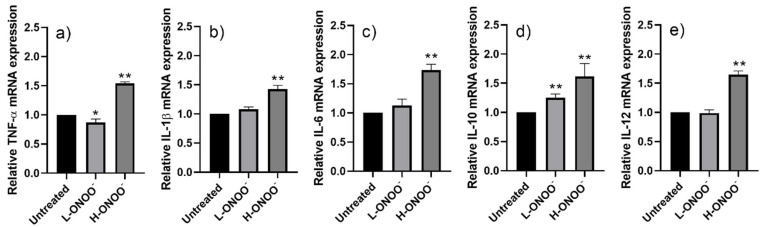
Relative mRNA expressions of (**a**) *TNF-α*, (**b**) *IL-1β*, (**c**) *IL-6*, (**d**) *IL-10*, and (**e**) *IL-12* in HUVECs after incubation with peroxynitrite at low dose (L-ONOO^−^) and high dose (H-ONOO^−^). * *p* ≤ 0.05 and ** *p* ≤ 0.01 vs. untreated HUVECs.

A low dose of peroxynitrite (L-ONOO^−^) did not kill HUVECs but inhited the mRNA expression of their respiratory antioxidant enzymes (e.g., *CAT*, *GPX1*, *SOD1*, and *SOD2*) (Figure 1). L-ONOO^−^ also inhibited the mRNA expression of the pro-inflammatory and anti-inflammatory cytokines *IL-6* and *IL-10* (Figure 2). On the other hand, significant changes in the targeted mRNA in the HUVECs were observed in the presence of a high peroxynitrite dose (H-ONOO^−^) that caused 20% cell death. H-ONOO^−^ further caused a 0.5-fold reduction in the expression of *CAT*, *GPX1*, *SOD1*, and *SOD2* mRNA (Figure 1) but upregulated the expression of inflammatory signaling cytokines (*TNF-α*, *IL-1β*, *IL-6*, *IL-10*, and *IL-12*) by about 1.5–2.5 times (Figure 2). The high peroxynitrite concentration (14.5 µM) was used to induce nitrosative stress in the HUVECs (ox-HUVECs).

Moreover, H-ONOO^−^ was introduced to HUVECs for up to 4 h and 24 h. However, the gene expressions of the antioxidant enzymes and inflammatory response cytokines of the HUVECS in the presence of H-ONOO^−^ for 4 h and 24 h were comparable to those of the normal cells (see Supplementary Material Figures S6 and S7). The results suggested that H-ONOO^−^ became inactive when the experimental periods were longer than 1 h. Then, nitrosative stress was applied to the HUVECs for 1 h.

### 3.3. Effects of Phytochemicals on HUVECs

**Figure 3:**
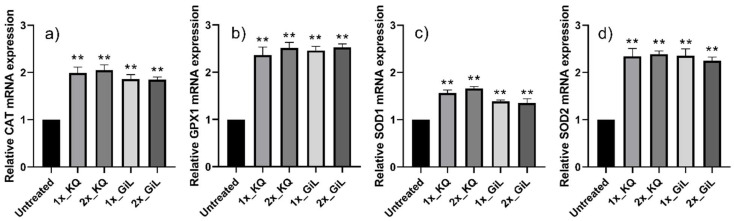
Relative mRNA expressions of (**a**) *CAT*, (**b**) *GPX1*, (**c**) *SOD1*, and (**d**) *SOD2* in HUVECs after incubation with the GiL extract at 62.5 µg/mL (1x_GiL) and 125 µg/mL (2x_GiL) and the kaempferol/quercetin mixture (1x_KQ and 2x_KQ) at equivalent concentrations to the GiL extract. ** *p* ≤ 0.01 vs. untreated HUVECs.

**Figure 4:**
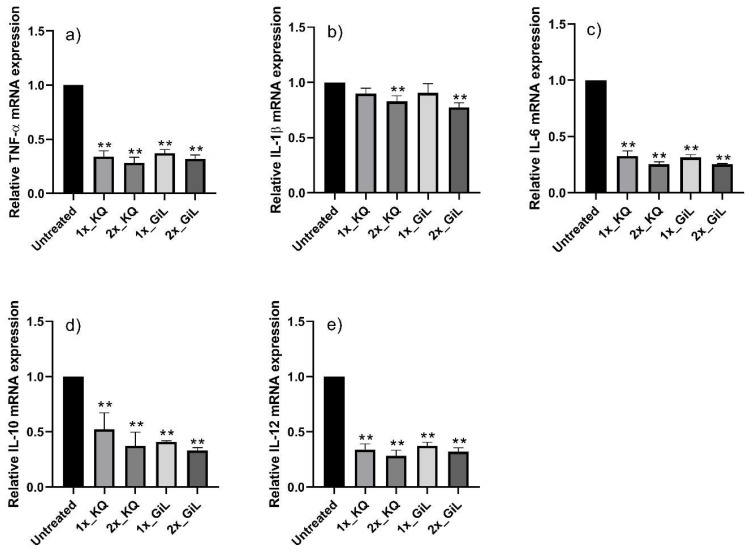
Relative mRNA expressions of (**a**) *TNF-α*, (**b**) *IL-1β*, (**c**) *IL-6*, (**d**) *IL-10*, and (**e**) *IL-12* in HUVECs after incubation with the GiL extract at 62.5 µg/mL (1x_GiL) and 125 µg/mL (2x_GiL) and the kaempferol/quercetin mixture (1x_KQ and 2x_KQ) at equivalent concentrations to the GiL extract. ** *p* ≤ 0.01 vs. untreated HUVECs.

The HUVECs treated with the GiL extract showed 2.0-, 2.4-, 1.5-, and 2.2-fold increases in their mRNA expression levels of *CAT*, *GPX1*, *SOD1*, and *SOD2*, respectively, compared with the untreated cells (Figure 3). However, the mRNA concentrations of *TNF-α*, *IL-1β*, *IL-6*, *IL-10*, and *IL-12* were downregulated 0.35-fold, on average, compared with the untreated HUVECs (Figure 4). Overall, the effects of the GiL extract on the HUVECs were correlated with the kaempferol and quercetin components in the extract.

### 3.4. Protective Effects of Phytochemicals on HUVECs Following Peroxynitrite Stress

**Figure 5:**
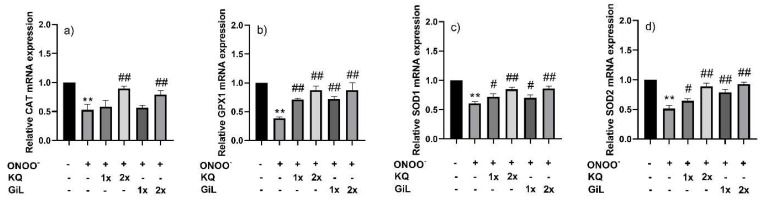
Relative mRNA expressions of (**a**) *CAT*, (**b**) *GPX1*, (**c**) *SOD1*, and (**d**) *SOD2* in HUVECs after pre-incubation with the GiL extract at 62.5 µg/mL (1x_GiL) and 125 µg/mL (2x_GiL) and the kaempferol/quercetin mixture (1x_KQ and 2x_KQ) at equivalent concentrations to the GiL extract, followed by peroxynitrite at a high dose (H-ONOO^−^). ** *p* ≤ 0.01 vs. untreated HUVECs, ^#^ *p* ≤ 0.05 and ^##^ *p* ≤ 0.01 vs. nitrosative cells (ONOO^−^).

**Figure 6:**
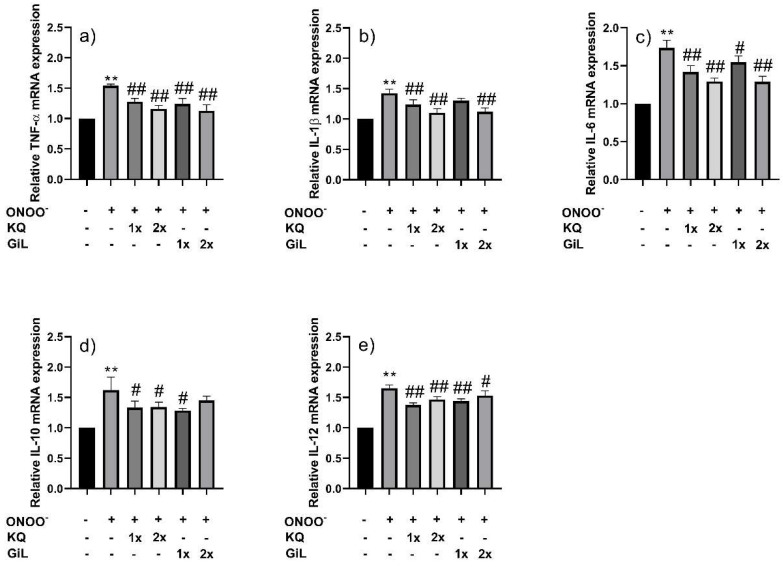
Relative mRNA expressions of (**a**) *TNF-α*, (**b**) *IL-1β*, (**c**) *IL-6*, (**d**) *IL-10*, and (**e**) *IL-12* in HUVECs after pre-incubating with the GiL extract at 62.5 µg/mL (1x_GiL) and 125 µg/mL (2x_GiL) and the kaempferol/quercetin mixture (1x_KQ and 2x_KQ) at equivalent concentrations to the GiL extract, followed by peroxynitrite at a high dose (H-ONOO^−^). ** *p* ≤ 0.01 vs. untreated HUVECs. ^#^ *p* ≤ 0.05 and ^##^ *p* ≤ 0.01 vs. nitrosative cells (ONOO^−^).

The viability of the HUVECs exposed to peroxynitrite was restored by pre-treatment with the GiL extract (See Figure S5 in Supplementary Material). The GiL extract protected the HUVECs by maintaining their cellular antioxidant enzymes (*CAT*, *GPX1*, *SOD1*, and *SOD2*) and inflammatory signaling cytokines (*TNF-α*, *IL-1β*, *IL-6*, *IL-10*, and *IL-12*) at levels comparable to those observed in normal HUVECs (Figure 5 and Figure 6); however, the protective effects of the GiL extract on the HUVECs were correlated with the concentrations of kaempferol and quercetin in the extract. Furthermore, the results indicate that the higher concentration of the extract seemed to have a greater effect on the gene expression of the HUVECs than the low concentration.

## 4. Discussion

Peroxynitrite induces oxidative stress in HUVECs. Nitrogen dioxide, carbonate radicals, superoxide radicals, hydrogen peroxide, and hydroperoxides are among the reactive nitrogen species that can be degraded to free radicals and intermediates through redox reaction cascades. One such species is peroxynitrite [30,31]. The mRNA expression of first-line enzymatic antioxidants (e.g., *CAT*, *GPX1*, *SOD1*, and *SOD2*) in cells is altered by these oxidative agents, which also influence cellular functions. These cellular antioxidant enzymes may catalyze the conversion of oxidative agents derived from peroxynitrite degradation into innocuous molecules (e.g., H_2_O_2_/alcohol and O_2_) [32]. Oxidative stress causes an imbalance in redox homeostasis and triggers the release of nuclear factor erythroid 2-related factor (Nrf2) from the Nrf2/KEAP1 complex in the cytoplasm. As a result, the Nrf2 moves to the nucleus and binds to the antioxidant response element (ARE). The Nrf2/ARE complex regulates the expression of gene clusters involved in mitochondrial protection and cellular antioxidant and anti-inflammatory defense [33,34]. Nrf2 can also activate DNA transcription to synthesize the mRNA of immune response cytokines via nuclear factor-κB (NF-κB) transcription factors [33,34,35].

A cellular immune response associated with oxidative stress was observed in HUVECs. The level of inflammation depended on the concentration of peroxynitrite. The lower dose of peroxynitrite used in this study triggered the minimal expression of pro-inflammatory cytokine mRNAs (*IL-1β* and *IL-6*) and activated the expression of anti-inflammatory signaling mRNA (*IL-10*). At this point, the HUVECs could still combat nitrosative and oxidative stress through cellular antioxidant systems; thus, the cellular inflammation was still controlled. A high dose of peroxynitrite weakened and partly killed the HUVECs. Additionally, the nitrosative and oxidative cells generated high levels of *TNF-α* and *IL-6* mRNA. These are characterized as chronic low-grade inflammatory signals [36] and cellular apoptosis markers [37,38]; thus, an increase in *TNF-α* and *IL-6* may cause the acute death of HUVECs, as observed in this study. Oxidative stress also affected the mRNA expression of an inflammatory response signal (*IL-12*) in innate immune cells [39,40,41]. The presence of *TNF-α*, *IL-6,* and *IL-12* mRNA could suggest early chronic inflammation in the vascular systems of individuals with diabetes, indicating the potential development of further diabetic complications [42,43]. *IL-12* is recognized as a biomarker for diabetic complications, particularly cardiovascular diseases. Controlling the *IL-12* levels in individuals with diabetes could mitigate the severity of diabetic complications [44].

The results of our investigation demonstrated that the studied GiL extract contained kaempferol and quercetin derivatives, which functioned as antioxidant/anti-inflammatory compounds, preventing nitrosative and oxidative stress in HUVECs. The free radical scavenging agents in GiL extract (kaempferol and quercetin) can detoxicate free radicals and intermediates produced during the degradation of peroxynitrite via HAT and SET [23]. The current study found that GiL extract can activate the mRNA expression of the cellular antioxidant defense system of HUVECs (e.g., *CAT*, *GPX1*, *SOD1,* and *SOD2*) at high levels. An increase in the mRNA levels of enzymatic antioxidants could be due to the Nrf2/ARE binding, resulting in the upregulation of the expression of genes encoding the enzymatic antioxidants [34].

In addition, the GiL extract inhibited the cellular inflammation induced by nitrosative and oxidative stress in HUVECs. Our results also showed that the observed decline in the mRNA expression of these cytokines (including *TNF-α*, *IL-1β*, *IL-6*, *IL-10*, and *IL-12*) was controlled by the kaempferol and quercetin content in the *G. inodorum* extract. Kaempferol and quercetin are plant flavonoids that can decrease the expression of immune response cytokine genes by suppressing inducible transcription factors such as the nuclear factor-κB (NF-κB) family. A reduction in NF-κB can downregulate the production of inflammatory cytokine mRNA [45,46].

Pre-incubating HUVECs with the GiL extract increased the antioxidant and anti-inflammatory benefits of HUVECs compared with cells pre-treated with kaempferol and quercetin. Our results suggest a synergistic effect of kaempferol, quercetin, and other natural compounds in the GiL extract [23,47,48,49].

Plant flavonoids with aglycone and glycoside structures can be absorbed through intestinal villi and into blood circulation after ingestion via several pathways [50,51]. Ounjaijean et al. [52] found that the consumption of GiL extract inhibited cardiac dysfunction in mice infected with *Plasmodium berghei*. Their study suggests that phytonutrients in GiL extract can be absorbed via the intestines and delivered to the bloodstream in humans. The ability of flavonoids in GiL extract to combat oxidative stress and the inflammation of vascular systems in vivo is promising.

This study has yielded promising results for the development of *G. inodorum* as an antioxidant and anti-inflammatory constituent to prevent vascular dysfunctions and diabetic complications. GiL extract can also provide additional anti-diabetic benefits, such as anti-inflammation [15,25,26], anti-insulin resistance, and insulin mimetic activity [15,53], the inhibition of *α*-amylase and *α*-glucosidase actions [19,20], a reduction in intestinal glucose absorption [20,21], and anti-adipocyte differentiation [54]. Thus, consuming *G. inodorum* leafy green vegetables can provide various phytonutrients that prevent diabetes and diabetic complications and support vascular health.

## 5. Conclusions

*Gymnema inodorum* leaf (GiL) extract was observed to exert antioxidant and anti-inflammatory effects against peroxynitrite-induced oxidative stress in vascular endothelial cells. The GiL extract modulated the upregulation of cellular enzymatic antioxidant genes (*CAT*, *GPX1*, *SOD1*, and *SOD2*) and helped to inhibit peroxynitrite-induced cellular inflammation by decreasing the mRNA levels of inflammatory signaling cytokines, such as *TNF-α*, *IL-1β*, *IL-6*, *IL-10*, and *IL-12*. The antioxidant and anti-inflammatory activities of the GiL extract were associated with its kaempferol and quercetin flavonoid contents.

This study demonstrates the protective effects of GiL extract against nitrosative stress-induced endothelial dysfunction, a major contributor to diabetes-related vascular complications. In human umbilical vein endothelial cells (HUVECs), the GiL extract upregulated the expression of cellular antioxidant enzymes (*CAT*, *GPX1*, *SOD1,* and *SOD2*) and suppressed the expression of pro-inflammatory cytokines (*TNF-α*, *IL-1β*, *IL-6*, *IL-10*, and *IL-12*). Moreover, the flavonoid contents of the GiL extract (kaempferol and quercetin) are responsible for its antioxidant and anti-inflammatory properties.

## Figures and Tables

**Table 1:** Information on genes and primer sequences used in this study.

Target Functions	Genes (Accession Numbers)	**Primer Sequences (5′-3′)**
Housekeeping gene	*GADPH* (NM_001289745.3)	F-5′-ACATCGCTCAGACACCAT-3′ R-5′-TGTAGTTGAGGTCAATGAAGGG-3′
Antioxidant enzymes	*CAT* (NM_001752.3)	F-5′-AGGGGCCTTTGGCTACTTTG-3′ R-5′-ACCCGATTCTCCAGCAACAG-3′
	*GPX1* (NM_000581.4)	F-5′-CCGGGACTACACCCAGATGA-3′ R-5′-CGTTCTCCTGATGCCCAAAC-3′
	*SOD1* (NM_000454.5)	F-5′-AGCATTAAAGGACTGACTGAAGG-3′ R-5′-GTCTCCAACATGCCTCTCTTC-3′
	*SOD2* (NM_000636.3)	F-5′-GTTGGGGTTGGCTTGGTTTC-3′ R-5′-ATAAGGCCTGTTGTTCCTTGC-3′
Inflammatory cytokines	*TNF-α* (NM_000594.4)	F-5′-CCCCAGGGACCTCTCTCTAA-3′ R-5′-TGAGGTACAGGCCCTCTGAT-3′
	*IL-1β* (NM_000576.3)	F-5′-TTGAGTCTGCCCAGTTCC-3′ R-5′-TTTCTGCTTGAGAGGTGCT-3′
	*IL-6* (NM_00600.5)	F-5′-ACAGGGAGAGGGAGCGATAA-3′ R-5′-GAGAAGGCAACTGGACCGAA-3′
	*IL-10* (NM_000572.3)	F-5′-GCCAAGCCTTGTCTGAGATG-3′ R-5′- GGCCTTGCTCTTGTTTTCAC-3′
	*IL-12* (NM_002187.2)	F-5′-GTCCTCAGAAGCTAACCATCTCC-3′ R-5′-CCAGAGCCTATGACTCCATGTC-3′

## Data Availability

Data is contained within the article. The original contributions presented in this study are included in the article/Supplementary Material. Further inquiries can be directed to the corresponding author(s).

## Supplementary Materials

The following supporting information can be downloaded at: https://www.mdpi.com/article/10.3390/antiox14040427/s1, Figure S1: Cell viability of HUVECs treated with peroxynitrite; Figure S2: Cell viability of HUVECs treated with GiL extract; Figure S3: Cell viability of HUVECs treated with kaempferol; Figure S4: Cell viability of HUVECs treated with quercetin; Figure S5: Cell viability of HUVECs pre-treated with GiL extract before exposing to peroxynitrite (ONOO^−^); Figure S6: Enzymatic antioxidant genes of HUVECs upon peroxynitrite stress (14.5 μM) for 1 h, 4 h and 24 h; Figure S7: Inflammatory response genes of HUVECs upon peroxynitrite stress (14.5 μM) for 1 h, 4 h and 24 h.

## Abbreviations

The following abbreviations are used in this manuscript:

GiL extract	*Gymnema inodorum* leaf extract
HUVECs	Human umbilical vein endothelial cells
mRNA	Messenger ribonucleic acid
*GADPH*	Glyceraldehyde 3-phosphate dehydrogenase housekeeping gene
*CAT*	Catalase gene
*GPX1*	Glutathione peroxidase 1 gene
*SOD1*	Superoxide dismutase 1 gene
*SOD2*	Superoxide dismutase 2 gene
*TNF-α*	Tumor necrosis factor-alpha gene
*IL-1β*	Interleukin-1 beta gene
*IL-6*	Interleukin-6 gene
*IL-10*	Interleukin-10 gene
*IL-12*	Interleukin-12 gene
NF-κB	DNA transcription factor; nuclear factor-κB
T2DM	Type 2 diabetes mellitus
ROS	Reactive oxygen species
AGEs	Advanced glycation end products
RNS	Reactive nitrogen species
HAT	Hydrogen atom transfer
SET	Single-electron transfer
PCA	Principal component analysis
HCA	Hierarchical cluster analysis
LPS	Lipopolysaccharide
HPLC	High-performance liquid chromatography
qRT-PCR	Quantitative reverse transcription-polymerase chain reaction
L-ONOO^−^	Low dose of peroxynitrite
H-ONOO^−^	High dose of peroxynitrite
KQ	Kaempferol and quercetin mixture
Nrf2	Nuclear factor erythroid 2-related factor
KEAP1	Kelch-like ECH-associated protein 1
ARE	Antioxidant response element
