# Supplementary material for: Antioxidant and Anti-Inflammatory Benefits of Gymnema inodorum Leaf Extract in Human Umbilical Vein Endothelial Cells Under Peroxynitrite Stress
Source: Antioxidants (Basel). 2025 Apr 1;14(4):427. doi: 10.3390/antiox14040427 (PMC12024022; doi:10.3390/antiox14040427)
Supplement: Supplementary file 1 [file antioxidants-14-00427-s001.zip › antioxidants-3539160-supplementary.pdf]

Supplementary Material

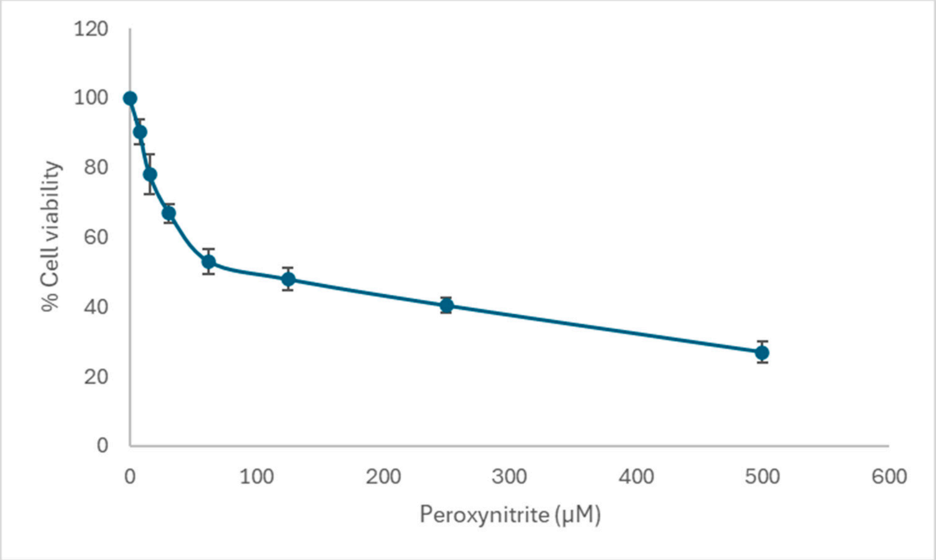

Figure S1. Cell viability of HUVECs treated with peroxynitrite.

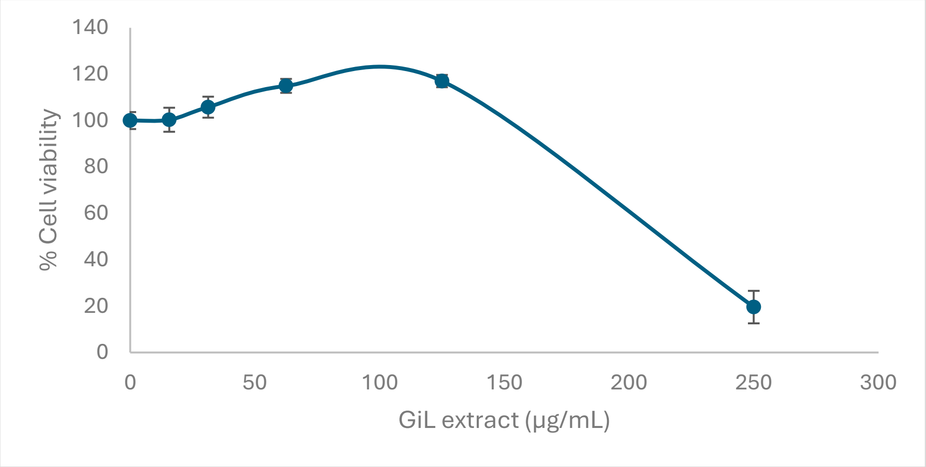

Figure S2. Cell viability of HUVECs treated with GiL extract.

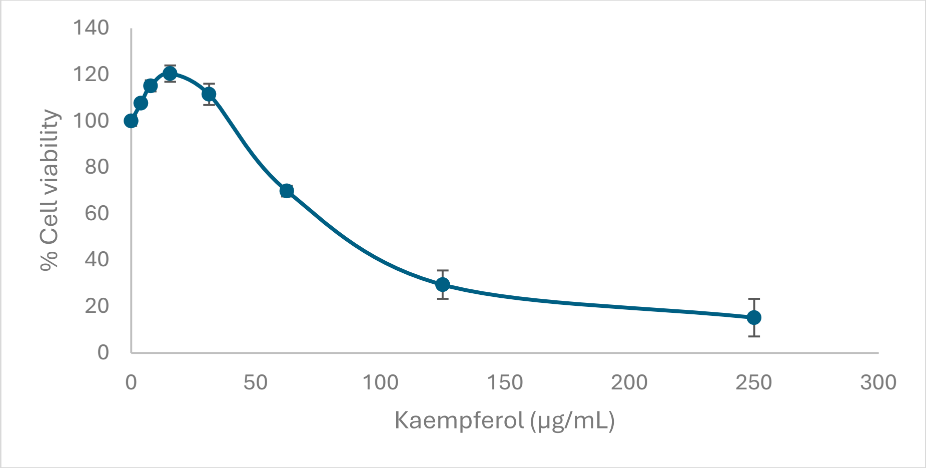

Figure S3. Cell viability of HUVECs treated with kaempferol.

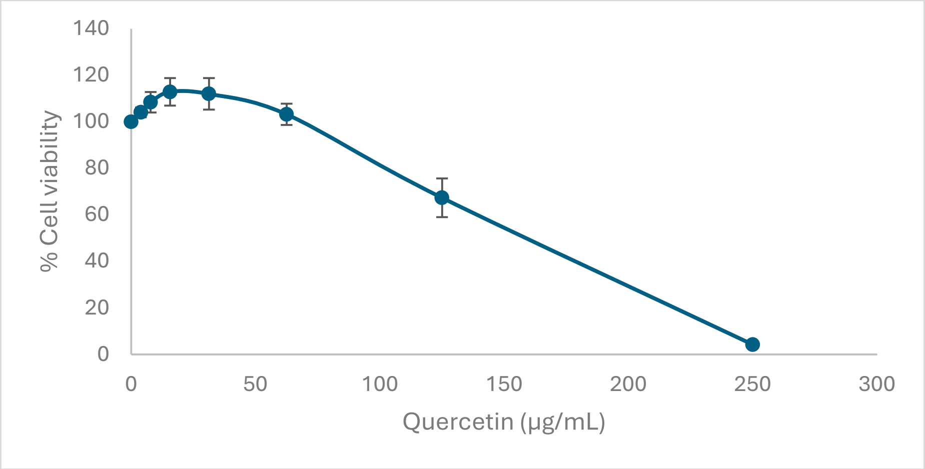

Figure S4. Cell viability of HUVECs treated with quercetin.

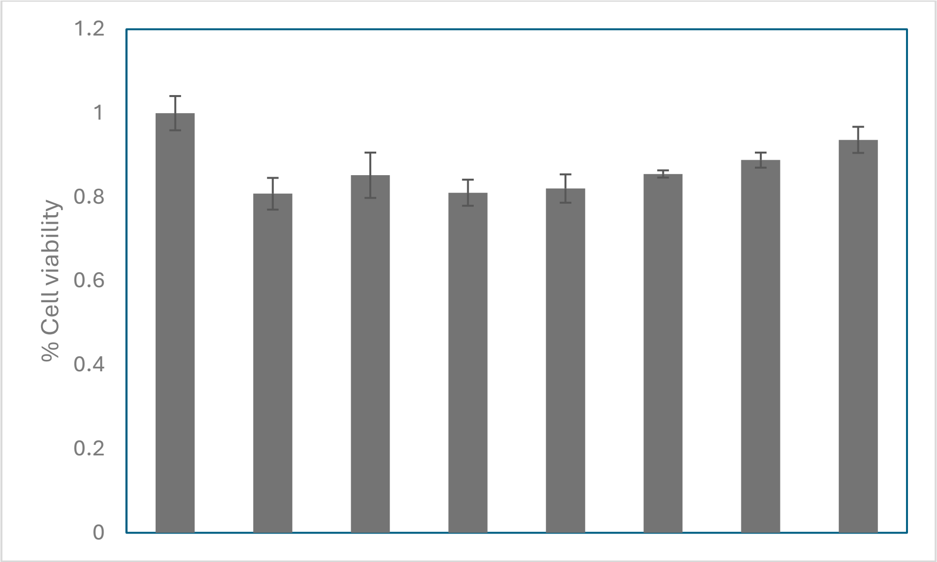

$ONOO^-$ (14.5 µM)	-	+	+	+	+	+	+	+
GiL extract (µg/mL)	0	0	3.9	7.8	15.6	31.2	62.5	125

Figure S5. Cell viability of HUVECs pre-treated with GiL extract before exposing to peroxynitrite ($ONOO^-$).

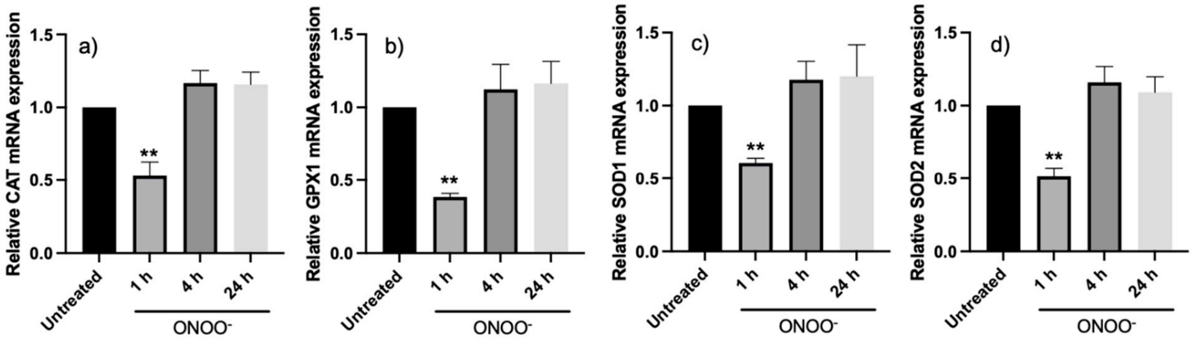

Figure S6. Enzymatic antioxidant genes of HUVECs upon peroxynitrite stress (14.5 µM) for 1h, 4h and 24h.

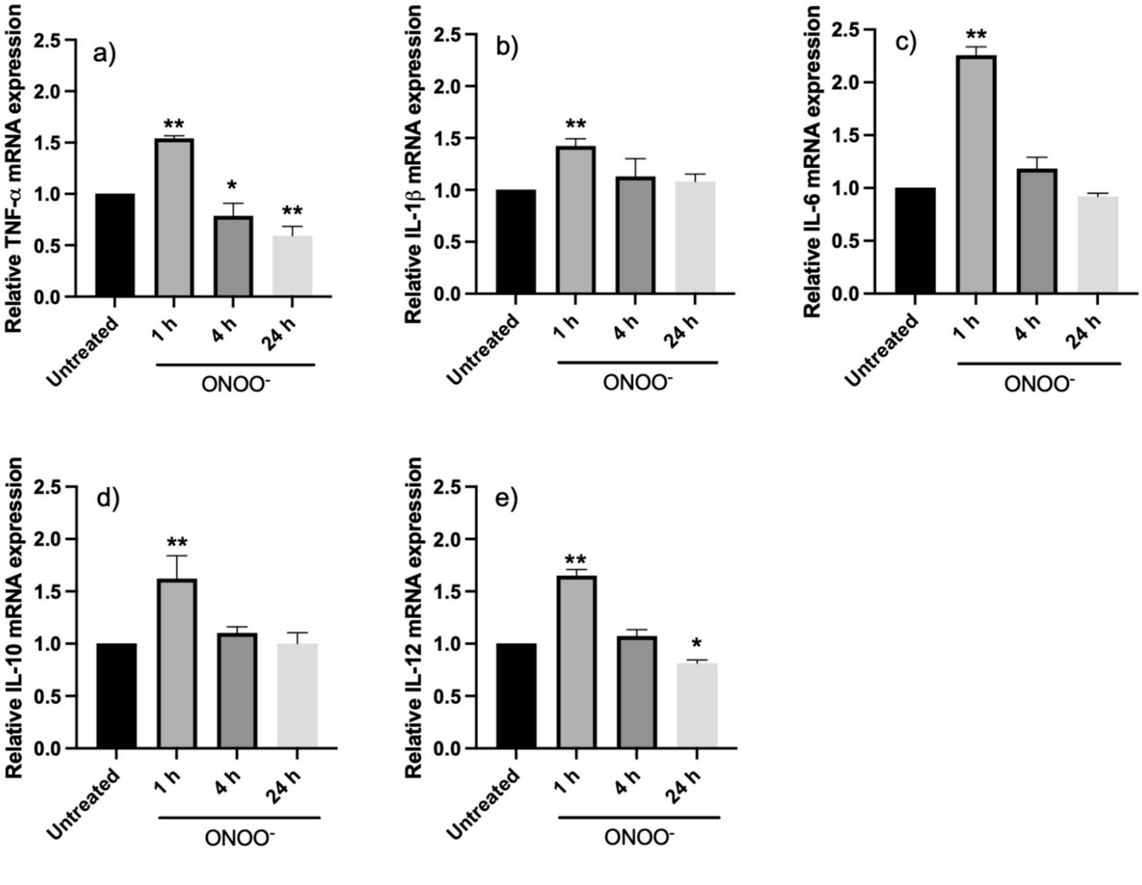

Figure S7. Inflammatory response genes of HUVECs upon peroxynitrite stress (14.5 μM) for 1h, 4h and 24h.
